# A new tool for early diagnosis of rheumatoid arthritis using combined biomarkers; synovial MAGE-1 mRNA and serum anti-CCP and RF

**DOI:** 10.11604/pamj.2020.36.270.21827

**Published:** 2020-08-12

**Authors:** Al-Qtaitat Aiman, Mwafi Nesrin, Albtoosh Amal, Al-Dalaien Nassar

**Affiliations:** 1 Department of Anatomy and Histology, Faculty of Medicine, Mutah University, Mutah, Jordan,; 2 Department of Biochemistry and Molecular Biology, Faculty of Medicine, Mutah University, Mutah, Jordan,; 3 Department of Orthopedic Surgery, Jordan University Hospital, The University of Jordan, Mutah, Jordan

**Keywords:** Rheumatoid arthritis, rheumatoid factor, synovial fluid, cyclic citrullinated peptides, MAGE-A

## Abstract

**Introduction:**

rheumatoid arthritis (RA) is a common autoimmune disease with unknown etiology and pathogenesis. Biomarkers have the potential to aid in the clinical diagnosis of the disease, or to provide means of detecting early signs of the disease. Evaluating Melanoma associated antigen genes (MAGE-1) mRNA expression rate in synovial fluid cells and serum levels of anti-cyclic citrullinated peptides (anti-CCP) and rheumatoid factor (RF) for RA early diagnosis.

**Methods:**

a total of 213 subjects were enrolled in the study, 135 RA patients and 78 normal subjects with traumatic knee joints (control group). Serum RF and anti-CCP were estimated quantitatively using ELISA. MAGE-1 mRNA expression rate was analyzed by RT-PCR.

**Results:**

a significant increase in serum levels of RF IgM and anti-CCP in RA patients compared to the controls. A positively significant correlation was found between serum anti-CCP and RF IgM. The expression rate of MAGE-1 mRNA was 100% in RA patients versus the controls (0%). The specificity and the sensitivity of the three biomarkers was 100%.

**Conclusion:**

the high expression rate of MAGE-1 in synovial fluid cells of RA patients is encouraging its utilization as a diagnostic biomarker for RA. The combined use of MAGE-1 transcript in synovial fluid cells, serum RF and anti-CCP is recommended for improving early diagnostic ability of RA.

## Introduction

Rheumatoid arthritis (RA) is a chronic progression disease causing joints erosion and systemic organs involvement in patients [1]. It affects about 1% of all ages and both sexes of the world’s population [2]. Progressive joints destruction and deformity with variable severity extent of the disease leads to the inflammation of synovial tissues [3]. According to ACR/EULAR 2010 classification criteria, the definite RA is based on synovitis manifestations in at least one human joint. The diagnostic items include: symptoms duration, serological and acute-phase response biomarkers abnormal levels and site of the affected joints [4]. Improvement of patients’ clinical status requires early diagnosis of RA. Screening tests should be used for assessing the disease progression, response to therapy and effectiveness of treatment. RA diagnosis in its early stage requires sensitive, specific, accurate and feasible biomarkers [5] that should be detected in blood and/or in the synovial fluid [6]. Some clinical biomarkers including anti-keratin antibodies, anti-cyclic citrullinated peptide (anti-CCP) antibodies, anti-perinuclear factor, anti-filaggrin antibodies, rheumatoid factors and others were used in RA diagnosis [7]. Rheumatoid factor (RF) is an antibody to the IgG Fc region [8]; it could be detected in different autoimmune diseases such as systemic lupus erythromatosus and RA. Furthermore, the pathogenic biomarkers, IgM and IgA RFs were also used to support RA diagnosis [9]. Moreover, cyclic citrullinated peptide antibodies are autoantibodies produced by the immune system that are directed against cyclic citrullinated peptides (CCPs). CCPs were used to measure peptidyl-arginine citrullination into peptidyl-citrulline [10].

Detection of anti-CCP antibodies has a predictive value for RA diagnosis [11], and early bone mineral density loss which occurs before the onset of the disease [12, 13]. Furthermore, anti-CCP antibodies biomarker has been added to the ACR/EULAR 2010 diagnostic criteria to support RA diagnosis and its classification [14]. On the other hand, melanoma associated antigen genes family (MAGE) are a group of genes located on X chromosomes encoding for large and highly conserved tumor antigens, sharing a common homology domain and associated to T-lymphocytes in different types of cancer [15]. They were originally described as completely silent in normal adult tissues, with the exception of male germ cells. Dozens of MAGE have been identified based on the differences in tissue-specific gene expression and gene structure. Their expression is not restricted to the reproductive tissues, but also in a wide variety of cancer types of diverse histological origin [16]. A recent report suggests their role in cancer progression [17]. The MAGE family has been divided into two big subfamilies: MAGE-I and MAGE-II. Type I was subdivided into three subfamilies (A, B and C). These subfamilies were almost found normally in testis, placenta and developing embryos. MAGE-A sub-family includes twelve related genes, one of them is a pseudogene gene [18]. The interaction between different MAGE-A proteins can perform complex different functions through potentiating specific oncogenic activities [19]. The expression of type 1 MAGE genes is cancer/testis specific; they are considered as cancer biomarkers and represent unique targets for cancer immunotherapies because of having antigenic properties and special expression pattern [20, 21]. Significantly, it was reported that MAGE antigen has a strong association with autoimmune diseases and the pathogenesis of the chronic inflammation in RA. Our study was designed to examine MAGE-I-mRNA expression rate in synovial fluid cells of RA joints, and serum levels of both RF and anti-CCP, and to evaluate their presence as early diagnostic biomarkers for RA in Jordanian patients.

## Methods

A total of 213 subjects were enrolled in this study from Orthopedic Surgery Outpatient Clinic, Jordan University Hospital, Jordan, in the period between November 2015 and December 2017. They were grouped into two groups: Group I (control group) comprised of 78 subjects (42 males and 36 females) with traumatic knee joint, age 26-54 years. Group II (RA group) included 135 RA patients (85 males and 50 females), age 32-57 years. An informed written consent was obtained from each patient and each subject of the control group, and the experiments were conducted according to the ethical forms approved by The Ethics Committee, Faculty of Medicine, Mu’tah University.

**Anti-CCP and RF assay:** blood samples were collected and stored at -80°C. Synovial fluids were aspirated in ethylenediaminetetraacetic acid (EDTA) tubes from the knee joints. Serum RF IgM and anti-CCP antibodies were estimated by ELISA using kits supplied by Diagnostic Automation/USA, in accordance with the methods of Dabadghao *et al*. [22] and Quinn *et al*. [23], respectively.

**MAGE-1mRNA assay:** total RNA was extracted from isolated synovial fluid cells using RNA-Spin total RNA extraction kit (South Korea/iNtRON Biotechnology), according to Chomczynski method [24]. RT-PCR was performed by one-step RT PCR-PreMix kit (South Korea/iNtRON Biotechnology). 8μl of ONE-STEP RT-PCR PreMix Kit were dispensed into PCR tubes, followed by addition of RNA templates and gene specific primers, then distilled water was added up to 20μl total volume and the components were mixed thoroughly. RT-PCR was carried out in a thermal cycler (Perkin Elmer Cetus, Norwalk, CT, USA): one cycle of two steps, reverse transcription reaction at 45°C for 30 minutes followed by denaturation of RNA: cDNA hybrid at 94°C for 5 minutes. Primers for both MAGE-1 and β actin genes were supplied by Integrated DNA Technologies/USA. Primers for MAGE-1 were [sense-(5/-CGG CCG AAG GAA CCT GAC CCA G-3/) (antisense-5/ GCT GGA ACC CTC ACT GGG TTG CC-3/)]. Primers for β actin to assess RNA integrity [25] were (sense-5/-GGC ATC GTG ATG GAC TCC G-3/ and antisense-5/-GCT GGA AGG TGG ACA GCG A-3/). PCR amplification of MAGE-1 and β actin genes was conducted using a thermal cycler following the method adopted by Khademvatan *et al*. [26].

The amplification was performed using 30μl of reaction mixture, containing 10μl of template DNA, 10pmol/μl of each primer, 10mM of dNTP mix, 50mM KCl, 10mM Tris-HCl, pH 9,25mmol/l of MgCl2, 10% dimethyl sulfoxide, 5U/8μl Taq DNA polymerase. The amplification cycle consisted of an initial denaturation cycle at 94°C for 30 seconds; followed by 35 cycles of denaturation at 94°C for 60 seconds, annealing at 65°C for 45 seconds, and extension at 72°C for 60 seconds; and a final extension cycle at 72°C for 10 min. Finally, 20μl of PCR products were run on a 1.5% agarose gel, then, stained and visualized under UV. A 100 bp DNA ladder was used to check PCR products and their sizes.

**Statistical analyses:** the obtained data was analyzed using SPSS 16 software package. All values were expressed as mean ± SD. Student's unpaired t test, Pearson’s correlation coefficient (r), and sensitivity and specificity tests (ROC) curve were applied p < 0.01 was considered significant.

## Results

The obtained results of RF and anti-CCP of the two groups of control subjects and patients are shown in (Table 1). There are significantly higher serum levels values of both RF and anti-CCP in the RA group compared to the control group (p < 0.001). Furthermore, the results obtained for anti-CCP serum levels and RF levels revealed a positive significant correlation in the RA group between the two variables (r=0.870, p < 0.001). The analysis of the sensitivity and the specificity of RF and anti-CCP for different cutoff points (54.29IU/ml and 12.16IU/ml for anti-CCP and RF, respectively) showed that both values were 100%. Furthermore, the area under the curve (AUC) for both biomarkers was 1.00. The expression rate for MAGE-1 was 100% in RA patients, while it was 0% in the controls. The expression of MAGE-1 was checked by RT-PCR electrophoresis of amplicons on 1.5% agarose gel, showed 421 bp bands of MAGE-1 in all RA patients.

**Table 1:** mean values ± SD of serum levels of RF and anti-CCP in RA and control groups

Parameter	Knee trauma subjects	RA patient	P
RF (IU/ml)	25.36 ± 3.12	116.79 ± 16.23	0.000
Anti-CCP (IU/ml)	2.64 ± 0.49	31.34 ± 5.63	0.000

* p<0.001 when the mean value of the RA group is compared to the control group

## Discussion

Environmental and genetic etiologies are implicated in RA pathogenesis affecting cartilages and bones of small and middle-sized joints [27]. The prevention of deformity, disability and irreversible damage of the influenced joints is expected by initial diagnosis and immediate effective therapy. The appropriate management of RA is required within 3-6 months of the disease onset to acquire an initial good disease prognosis [28]. Identification of different combinations of biomarkers are recommended for facilitating and improving RA early diagnosis [29]. As shown above, MAGE-I-mRNA expression rate in synovial fluid cells from RA patients was 100% compared to the traumatic knee joints of normal subjects (0%). Those findings are compatible with the data obtained by Abd Elsalam *et al*. [25] and McCurdy *et al*. [21]. The latter explained the expression of MAGE-A1 transcript by the mononuclear cells from the inflamed joints of juvenile RA patients. In spite of all the similarities in the results obtained in our study and the results reported in the study of Abd Elsalam *et al*. [25], however, there are certain differences between the two studies, including the number of the enrolled subjects and their different origins.

In our study, a total of 213 subjects were enrolled in the study compared to only 30 subjects in their study. Furthermore, the adopted methods for the amplification of MAGE-1 and β actin genes were entirely different between both studies. On the other hand, it was reported that there is a correlation between MAGE-A gene expression and the poor prognosis of epithelial ovarian and lung cancers suggesting not only its diagnostic role but also its role as a prognostic biomarker for the treatment and effective follow up [30, 31]. Moreover, MAGE-A1 gene is playing an important role in the inflammation process, which could be attributed to T cells activation via immune response system activation [32], consequently enhancing cytokines up-regulation and providing widespread activation of the immune response [20]. Our obtained results revealed significant increase in RF and anti-CCP serum levels in RA patients compared to the controls, which correspond with other previous studies [29, 33, 34]. Anti-CCP and RF are considered as a part of natural immunity [35], and detected in the serum of healthy subjects before the progression of RA [36]. As shown above, there is a significant positive correlation between RF and anti-CCP levels, which is also compatible with other studies [25, 37, 38]. RA progression is highly associated with serum levels of anti-CCP antibodies and affected by various copies of HLA-DRBI genotypes. Serum levels of anti-CCP antibodies was ten folds higher in HLA-DRBI*04 alleles positive patients compared to HLA-DRBI*04 alleles negative subjects [37].

In addition, the results of the present study showed that the specificity and the sensitivity for the three biomarkers was 100%. Previous report showed that the specificity of anti-CCP, RF-T, RF-IgM, RF-IgA and RF-IgG were 95.3, 82.3, 97, 98.9 and 99.4, respectively [39]. The same report showed that the specificity for all RF isotypes was higher than the specificity of anti-CCP, in contrast to their sensitivity, which was lower. Early identification of patients with RA will help improve clinical outcomes with early treatments. Therefore, the analysis of the biomarkers of disease activity and severity are needed. For many diseases, a single biomarker can be informative on a population level but not at the level of the individual patient [28]. This inadequacy has shifted attention to the use of multiple biomarkers analysis. The sensitivity and the specificity of the combined three biomarkers (anti-CCP1, anti-CCP2 and RF) assay in the same groups were shown to be in a less range than the sensitivity and the specificity of anti-CCP2 assay alone [40]. Although, previous studies reported inconsistent results to ours concerning the sensitivity and the specificity of anti-CCP and RF [41, 42]. Both biomarkers (anti-CCP and RF) are considered prognostic indicators of RA, the higher the level, the poorer the prognosis and the earlier the erosion of the affected joints. RF is associated with the disease activity, while, anti-CCP antibody is a highly specific biomarker for RA diagnosis [43, 44].

The sensitivity, the specificity and the accuracy of combined detection of anti-CCP antibody and RF for RA diagnosis were 90.2%, 83.3% and 87%, respectively [40]. Similarly, anti-CCP accuracy for RA diagnosis was shown to be better due to its highly positive and negative predictive values [45]. Therefore, the two biomarkers should be complementary because some patients are anti-CCP positive but RF negative [46]. On the other hand, the third generation of anti-CCP antibodies can be used to distinguish between RA and non-RA patients [47]. Besides, the positivity of CCP3 and CCP2 can help in recognizing RA patients and prediction of joints erosion [48]. In the present study, the variations with other reports regarding the sensitivity and specificity of RF and anti-CCP can be attributed to many factors, for example, whether the enrolled patients were under treatment or not, RA status whether it is active/not active or advanced/not advanced, besides the racial and geographical differences [28].

## Conclusion

Serum levels of RF and anti-CCP are higher in RA patients than the controls. The correlation between RF and anti-CCP levels is significantly positive. Also, MAGE-1 transcript expression rate was 100% in all enrolled RA patients, providing a diagnostic and prognostic tool for the disease. The combination of these three biomarkers can be used for facilitating early diagnosis of RA. Further prospective studies of early RA on a larger sample of patients are required to determine the predictive ability of MAGE-1 transcript expression rate and understanding its role in chronic inflammation of RA and immune response development.

### What is known about this topic

- Rheumatoid arthritis (RA) diagnosis in its early stage requires sensitive, specific, accurate and feasible biomarkers. To find specific biomarkers will benefit both RA patients to find relief from the disease and physicians to monitor the disease development;
- Several studies have revealed RA-related biomarkers including rheumatoid factor (RF), anti-cyclic citrullinated peptide (anti-CCP) antibodies;
- Melanoma associated antigen genes family (MAGE) are a group of genes located on X chromosomes. They were expressed in a wide variety of cancer types and has a strong association with autoimmune diseases and the pathogenesis of the chronic inflammation in RA.

### What this study adds

- Serum levels of RF and anti-CCP are increased in RA patients with appositive correlation between the two biomarkers;
- MAGE 1 transcription expression rate in synovial fluid cells of RA patients was 100% compared to the traumatic knee joints of normal subjects (0%);
- The combined use of these three biomarkers is recommended for early diagnosis of RA.
